# Samuel Thomas Von Soemmering's History of the Viscera and the Organs of Sense in the Human Body

**Published:** 1845-10

**Authors:** 


### ART. X.

*Samuel Thomas von Soemmering, Lehre von den Eingeweiden und Sinnesorganen des menschlichen Körpers.* Umgearbeitet und beendigt von G. HUSCHKE.—*Leipzig,* 1844.

*Samuel Thomas von Soemmering's History of the Viscera and the Organs of Sense in the Human Body.* Edited and completed by G. HUSCHKE.—*Leipsic,* 1844. 8vo, pp. 950.

THIS is the fifth volume of what is called the new edition of Soemmering's great anatomical work 'Vom Baue des menschlichen Körpers.' The name is rather inappropriate, seeing that in all the seven volumes there is not above one page in a hundred in which Soemmering could recognize a passage of his own. It is neither more nor less than a great and admirable system of anatomy worked out by Bischoff, Henle, Huschke, Theile, Valentin, Vogel, and R. Wagner; each of whom has taken that department for which his chief studies peculiarly fit him, and of whom all those whose volumes have yet appeared seem to have entered into a honorable rivalry for preeminence. The result has been the production of a series of anatomical works with which, taken as a whole, none yet published can compete. The merits of the several volumes that were published previous to this are different; but in kind rather than in degree. Those of Theile on the Muscular and Vascular Systems are models of treatises in practical anatomy; every description appears to have been written in the dissecting-room, and to have been again and again compared with fresh dissections. There is besides, a genuine dryness about them, characteristic of the sound practical anatomist; and yet in the very dryness a tone of enthusiasm and of love of certainty, such that one might vow the writer went to his work with a thorough affection for it. Time after time, when other books, good systems too, and monographs, have been unclear or silent, Theile has told us all the truth; and not less often has he indicated things repeatedly overlooked, yet certainly to be found if only looked for in his fashion.

The merit of Valentin's volume on the Nerves is scarcely less. In most parts, especially in all that relates to the descriptive anatomy of the cranial nerves, it leaves very little to be said; its minuteness of description is carried to the very furthest point, and, with an apparent contempt for degrees of importance, filaments hardly visible are honoured with half-pages for their histories. All this merit of minuteness it has pre-eminently; but in its tediousness of style, its frequent obscurity, its divisions and sub-divisions of chapters, sections, paragraphs, its vain pretences of indications and references by all the forms of numerals, signs, and letters, Greek, Arabic, and Roman—it is overwhelming. Happy is he who has no need to study more than one page in one long sitting.

The contrast is complete between Valentin, and either Bischoff or Henle. The volume of the former on Development, and that of the latter on General Anatomy, are certainly, both in style and in contents, nearly faultless. All that is known on either subject they tell in the most agreeable form and manner.

This present volume by Huschke is the first of the series which appears to us to possess not only a different kind, but a less degree, of merit than the others. Long it is, and dry, very dry; but its dryness is not, like that of Theile's volumes, from simple accuracy and minute detail, nor, like Valentin's, from the descriptions of things exceedingly and almost uninterestingly minute; but rather because things quite obvious and commonplace, external shapes and outlines, and old-fashioned names and notions, and weights and measures, and mere sketches of superficial and imperfect physiology, are introduced in unnecessary profusion, while much of really minute anatomy and good modern physiology is omitted. We read on, page after page, and not a passage arrests attention; as for the anatomy, we feel as if we had read it nearly all before, in Haller or Malpighi, or for some of the best of it, in Hildebrandt or Cloquet; and, for the physiology, it is like that of answers to examination papers, thin, slippery, and incomplete.

The exceptions to this rule, the parts of the volume which are really new, are not such as to alleviate the faults. They consist chiefly of the absolute and relative weights and measures of the several organs in children of various ages under eight or ten years old, and in fœtuses near the end of gestation. With these also are many new estimates of the dimensions and weights of viscera, &c. in the adult. Thus:

"The kidneys of the new-born child, though absolutely much lighter than in the adult, are yet in proportion to the whole body much heavier, inasmuch as the weight of the two kidneys is to that of the whole body of the infant, as 1: 82—100, in the adult in the relation of 1 :225. They therefore do not grow uniformly with the rest of the body, although the secretion of urine becomes more energetic after birth. The absolute weight of a kidney in the new-born child is 6—10—15 grammes [French? of 15·5 grains each?] in a child 7 or 8 days old, the left weighed 20, the right 18 grammes; in one 3 weeks old, the left 13, the right 12 gr.; in one 6 weeks old, the left 17, the right 15 gr.; in one half a year old, the left 16·95, the right 15·5 gr.; in a girl 2 years old, the left 31, the right 28 gr; in a boy 3 years old, the left 43, the right 44 gr.; in one 3½ years old, the left 49, the right 55 gr; in a girl 6 years old, the left 48, the right 41 gr.; in a boy 8 years old, the left 80, the right 68 grammes." (pp. 345-6.)

Such is the character of what is especially new in this book. Facts of this kind give it value; they are not to be found elsewhere, and they may, some day, be usefully employed; but they do not compensate for the absence of facts of more interest than they themselves possess.

Similar facts to those just quoted are collected for nearly all the important viscera. We cannot quote them all, but for further examples, as well as because any truths relating to these organs are rare and interesting, we will give the general summary of what in this regard is said concerning the vascular glands.

"The absolute weight of the spleen, and its weight in proportion to that of the whole body increase rapidly after birth; and its proportionate weight soon attains its highest standard, so that in the adult it has no decidedly greater proportion to the body than it has after birth; nay, in some cases it even decreases. It varies between 1:235 and 1:240. But its relation to the weight of the liver is proportionally greater in the adult than in the infant. Before and within three days after birth, the weight of the spleen was found to bear proportions to that of the liver, varying between 1:49 and 1:12·8; but in six adult men the proportions were, in three of them as 1:6; in one as 1:10; and in two, as 1:11......I found moreover, in these cases, an increase of weight in nearly an equal proportion to the increase of the body, especially in the first periods after birth." (p. 185.)

"After birth the thyroid gland diminishes. I found in the new-born child its proportion to that of the body to be as 1:400—243; after three weeks, the proportion was 1:1166; and in the adult as 1: 1800. Its vessels also decrease after birth, as Meckel first observed. In old age its tissue becomes harder, &c." (p. 299.)

"The development [?] of the thymus gland after birth, is in so far remarkable as it is one of the few organs which die after birth. It belongs almost exclusively to the fœtus and the first periods of childhood. In the mature fœtus it weighs about 4 drachms; but in the child it weighs only 1. It goes on indeed to grow after birth; but, in proportion to the rest of the body it decreases. I found its weight in proportion to that of the body, in two fœtuses of eight months, as 1 : 221·428, its weight being 2·8 grammes....in a mature male fœtus as 1: 358·49, (its absolute weight 53 grammes); in a girl just born as 1 : 220·461, (its weight 13 grammes); in a boy 3 days old, as 1: 340, (its weight 5 grammes); in one 9 days old, as 1: 554, (its weight being 2·7 gr.); in one 3 weeks old, as 1: 675, (its weight 3·6 gr.). Hence it is evident that its proportionate weight rapidly diminishes after birth. And soon it also diminishes absolutely. In a girl, 6 years old, it weighed only 1·45 grammes. . . . . . Its specific gravity also gradually diminishes. It diminishes from below upwards; in the opposite direction, therefore, to that in which it is developed." (p. 305.)

"Even in the fœtal state, towards its conclusion, the renal capsules gradually diminish; and this decreasing proportion continues after birth. In new-born children I found them weigh about from 36 to 72 grammes; and their proportion to the weight of the body was between 1 : 475 and 1 : 705; while in the adult it is between 1: 4800 and 1 : 10800. It is further known that in the fœtus the renal capsules are large in proportion to the kidneys. In eight months' fœtuses I found the weight of the former to that of the latter to be as 1 : 2 or 3; in new-born children as 1: 3 or 4; after four weeks as 1 : 6; and in the adult as 1 : 14 or from that to 1 : 30. And this holds also in individual cases; so that the heavier the kidney the lighter in general is the adjacent renal capsule; and *vice versa.*" (p. 361.)

